# A novel skin cancer prevention strategy: Preservice teachers' perceptions of a sun safety intervention and experiences in schools

**DOI:** 10.1002/hpja.638

**Published:** 2022-07-21

**Authors:** Joseph J. Scott, Robyn S. Johnston, Jill Darby, Sally Blane, Mark Strickland, Bronwen M. McNoe

**Affiliations:** ^1^ School of Education and Tertiary Access University of Sunshine Coast QLD Australia; ^2^ School of Education Edith Cowan University WA Australia; ^3^ Telethon Kids Institute and University of Western Australia WA Australia; ^4^ School of Medical and Health Sciences Edith Cowan University WA Australia; ^5^ Cancer Council Western Australia WA Australia; ^6^ Social and Behavioural Research Unit University of Otago New Zealand

**Keywords:** children, perceptions, prevention, school, skin cancer, sun safety, teacher education

## Abstract

**Issue addressed:**

Teachers play a vital role in developing children's sun protection routines however upskilling preservice teachers (PSTs) while at university has not yet been trialled as a targeted skin cancer prevention strategy. Hence, this study investigated PSTs perceptions and experiences of sun safety following a brief pilot intervention and placement in primary schools in Western Australia.

**Methods:**

This study used a triangulation mixed methods design. Participants (n = 161) completed a post intervention survey which was analysed quantitatively. A random sub‐sample was invited to participate in focus groups (three groups, n = 21) and one‐on‐one interviews (n = 4). This data was transcribed and uploaded in NVIVO software for thematic analysis.

**Results:**

Participants felt the intervention increased their awareness of the dangers of overexposure to ultraviolet radiation (UVR) with many feeling more knowledgeable, skilled and confident to teach sun safety in school settings. Most reported clear sun safety messages in their placement schools. However, only 34.4% reported they had been briefed on the school's sun safety procedures. There was consensus among PSTs that sun protection in primary schools needs to be improved to maximise the protection of children from harmful UVR overexposure. Participants supported a need for consistent sun protection messaging across primary schools with greater emphasis on education rather than compliance management to sun protection.

**Conclusion:**

Enhancing existing teacher education programs to include more rigorous curriculum content and pedagogical approaches to sun protection education is a novel skin cancer prevention strategy and could feasibly support PSTs self‐efficacy to effectively deliver sun safety curriculum in Australian schools.

## 1 INTRODUCTION

Skin cancer (melanoma and keratinocytic cancers) is one of the most commonly diagnosed cancers globally^1^ with incidence rates continuing to increase.^2^ Australia has one of the highest incidence rates of skin cancer in the world^1^ with one in three individuals developing some form of skin cancer by age 70.^3^ Research has consistently concluded that exposure to harmful ultraviolet radiation (UVR) from the sun significantly increases the risk of developing melanoma and keratinocytic cancers in later life.^4^, ^5^ The ultraviolet index (UVI) is a simple measure of the UVR level at the earth's surface and can be used as an indicator for the risk of skin damage^6^ and hence is now commonly used as a health promotion tool.^7^

Protecting children's skin from harmful UVR exposure has been reported from an epidemiological perspective as one of the most effective ways to prevent future skin cancer development.^5^The World Health Organization has advised it is particularly important that children are protected from harmful UVR exposure as, compared to adults, their skin is thinner and more sensitive to sunburn.^8^ For children, their educational and recreational activities typically coincide with peak UVR times.^9^ As primary (elementary) school teachers have key responsibility for children during peak UVR hours during weekdays, it is prudent to target teachers to advocate for sun safety health promotion.^10^ Hence, primary schools are a particularly important setting for sun safety education programs.^11^

However, across Australia, school‐based UVR education, sun safety programs and approaches to sun protection are inconsistent.^12^, ^13^ Teacher education has been identified as key in the implementation quality of school‐based health programs.^14^ Targeting preservice teachers (PSTs) (teachers‐in‐training) while they are at university during their initial teacher education degree is novel and could be a potential strategy to create and improve robust whole school approaches to sun protection in primary schools. However, there is a paucity of research on whether teachers are adequately trained or equipped to educate their students on how to use the UVI and effectively implement sun protection practices. Given primary school teachers' potentially influential role in child sun protection and other health behaviours,^10^ there are strong benefits to developing teachers' skills and understandings related to teaching and enabling students' daily sun protective practices.

Understanding the UVI in a particular setting is a critical component of this. However, research suggests understanding of the UVI remains limited in the general Australian population,^15^ and this is likely to also be the case in PST populations.

Providing PSTs with deep content knowledge about UVR and the UVI could lead to more effective whole school health promotion and related curriculum implementation. As school‐wide approaches to sun safety education are recommended over isolated classroom activities,^16^ it is important that all teachers and school staff have the knowledge and skills to empower young people to protect themselves from the sun. PSTs could play an important role in improving the future of sun safety in schools, however, little is known regarding their perceptions and experiences of school‐based sun safety. Enriching existing teacher education programs to include more rigorous curriculum content and pedagogical approaches to sun protection education could be a potential strategy to support PSTs self‐efficacy to deliver sun safety in schools. Therefore, this study addresses this gap in the literature and aims to explore PSTs perceptions of a sun safety education intervention and explore their attitudes towards tanning, sun protection and experiences of sun safety approaches in primary schools while on placement. Findings from this study provide important information to inform university‐based interventions for PSTs.

## 2 METHODS

### 2.1 Participants and procedures

Approval to conduct this research was granted by the relevant University Human Research Ethics Committee (HREC22500). All participants provided informed consent to participate in the study. The sample was drawn from a larger study by Scott et al^17^ and included second‐ and fourth‐year undergraduate PSTs enrolled in the Bachelor of Primary Education in 2019 at one Western Australian university. The sample group both attended an interactive short 45 min sun safety education workshop (intervention) delivered by the research team and after they completed their normal school‐based placements as required for their degree, they completed a short survey. A sub‐sample of participants were randomly selected from this group and invited to participate in semi‐structured face‐to‐face focus groups or interviews (see Table 1). Those unable to attend face‐to‐face were offered to participate via telephone interview. Consistent protocols were followed during interviews and focus groups to ensure reliability.

**TABLE 1:** Characteristics of larger and focus group samples (n = 151)

Preservice teacher participant demographics	Larger sample N (%)	Focus group and interviews N (%)
Student year group		
Second‐year student	120 (79.5)	20 (80)
Fourth year student	29 (19.2)	5 (20)
Missing	2 (1.3)	
Age (mean: 23.21 years; median: 20 years)		
<24 years	115 (76.2)	19 (76)
25‐29 years	19 (12.6)	3 (12)
30+ years	17 (11.2)	3 (12)
Gender		
Males	30 (19.9)	6 (24)
Females	121 (80.1)	19 (76)
Country of birth		
Australia	117 (77.5)	21 (84)
Other	34 (22.5)	4 (16)
Cultural background		
Australian	96 (63.6)	19 (76)
Other: (please specify)	54 (35.7)	6 (24)
Prefer not to say	1 (0.7)	0 (0)
Language spoken at home most frequently		
English	145 (96)	25 (100)
Other	6 (4)	0 (0)
Schooling in Australia		
Primary only	1 (0.7)	0 (0)
Secondary only	10 (6.6)	1 (4)
Both primary and secondary	131 (86.7)	23 (92)
Neither, I attended outside Australia	9 (6)	1 (4)

#### 2.1.1 Intervention

The preservice teacher sun safety (PSTSS) intervention was an exploratory pilot to inform a large study and comprised of a face‐to‐face interactive workshop conducted by members of the research team in March 2019. The social cognitive theory (SCT)^18^ and the social ecological model (SEM)^19^ where used to inform the design of the intervention. The SCT provided a framework for exploring interactions between sun behaviours, attitudes and environmental influence and self‐efficacy. The SEM was used to explore determinants that influence the adoption of UV protective behaviours across individual, organisation and community levels. The intervention focused on sun safety education, sun protection strategies for primary‐aged children, school sun protection policies and availability of resources for teachers including the UVI and Cancer Council's SunSmart smartphone application which provides daily UVR levels and times of day where sun protection is required.^20^ The intervention details are described in Table 2.

**TABLE 2:** Description of the intervention

Intervention	Preservice teacher sun safety (PSTSS) intervention
Target population	Preservice teachers at one Australian University
Sample	Survey completion (n = 161) 3 x Focus groups (n = 21) 4 x One‐on‐one interviews (n = 4)
Theoretical Framework	Social cognitive theory^a^ (Bandura^18^) Social ecological model^a^ (Bronfenbrenner^19^) Theoretical domains framework^b^ (Cane et al^21^)
Data collection timeframe	Baseline: March 2019 Intervention: March 2019 Follow‐up: May 2019^c^
Type	Interactive educational workshop (multiple large group workshops delivered across 3 university campus sites)
Duration	45 min
Delivery	Members of the research team and staff from Cancer Council WA
Content	Sun safety education, effective sun protection measures for primary‐aged children, school sun protection policies, availability of resources for teachers including the UV Index and Cancer Councils SunSmart smartphone application
Format	Informal interactive (practical and theory) workshop format with use of PowerPoint presentation slides, interactive activities quizzes, props and teaching resources. Participants explored the use of sun protection measures/tools (different hat types, sunglasses, ways to seek shade when teaching outside, school uniforms and clothing), online UV indexes, weather forecasts and the SunSmart smartphone application (Cancer Council, 2010)

^a^ Use as framework to inform intervention design.

^b^ Used as framework to inform design of focus group questions.

^c^ Follow‐up 6‐wk postintervention used to allow time for PSTs to complete placement.

#### 2.1.2 Survey

Participants completed a brief survey 6 wk postintervention. Six weeks was selected as this allowed sufficient time for completion of respective placements in schools. Participants were emailed a QR code and link to the online survey. Questions related to: participants' attitudes towards sun protection and their in‐school experiences of sun safety while on placement (three questions, 5‐point Likert scale anchored at strongly disagree and strongly agree); and PSTs sun safe behaviours while on placement (two questions; 5‐point Likert scale anchored at never and everyday) (see Table 3).

#### 2.1.3 Focus groups and interviews

The theoretical domains framework^21^ was used to inform the design focus group questions exploring areas including perceived knowledge, skills, social role/identity, beliefs about capabilities, intentions, and social and environmental influences on behaviours and perceptions. Questions focused on participants' personal tanning and sun behaviours as wells as their attitudes towards, and experiences with, school‐based sun safety while on placement. On completion of the survey, a random sample of survey participants were invited by phone to participate in focus groups. To ensure consistency, the same researchers (J.S. and R.J.) conducted both the interviews and the focus groups. Researchers were not known to the study participants. Focus group included between five and nine participants and lasted approximately 40 min and interviews approximately 20 min. Each focus group/interview was audio recorded, with permission, and later independently transcribed verbatim by a research assistant outside the research team. After three focus group interviews and four interviews, J.S. and R.J. agreed that data saturation had been achieved,^22^ therefore, no further invitations were extended.

### 2.2 Analysis

This study used a triangulation mixed methods design to guide analysis of data.^23^ Survey data was downloaded from Qualtrics XM Platform and imported into IBM SPSS Statistics 27 (SPSS, Inc.) software for quantitative analysis (alpha set at *p* < 0.05). Descriptive analyses were used to assess frequency and mean data. Focus group and interview transcriptions were uploaded in NVIVO 12 software (QSR International) for analysis. Thematic analysis was completed to generate themes.^24^, ^25^ Researchers J.S. and R.J. independently reviewed one interview and one focus group transcript to generate initial codes and then discussed consistencies and discrepancies between identified codes. Codes were combined and refined at this time. Both coders then independently coded the remaining focus groups and interviews, before reviewing coding, with inter‐coder reliability analysis revealing substantial agreement (*k* = 0.61). Although there were minimal differences in coding; some codes were reviewed, refined and re‐coded where appropriate. Once all transcripts were coded, key domains and their respective dimensions were thematically analysed.

## 3 RESULTS

### 3.1 Sample

Of the 161 participants, 10 did not provide responses to survey questions focusing on their experiences in schools and were excluded from the analysis. The demographics of study participants (n = 151) are provided in Table 1. Participants were aged between 18 and 54 years (mean = 23.3, SD = 6.64) and the majority were female (80.1%), Australian‐born (77.5%), spoke English at home (96.0%) and attended both primary and secondary school in Australia (86.8%). The focus group and interview participants shared similar demographic characteristics to the larger sample.

### 3.2 Survey

Data indicated that most PSTs wore a hat when outdoors on placement every day (55.0%) or often (27.2%) (Table 3). Sunscreen use was much less common than hat use with most reporting that they never used it, used it rarely, or used it sometimes (27.8%, 22.5% and 21.2% respectively). Less than one fifth (18.0%) of participants reported that they wore sunscreen everyday while on placement. When reflecting on their experiences of their last placement, only 34.4% agreed/strongly agreed they had been briefed on sun safety procedures in the school, with a further 23.2% reporting a neutral response to this question. Most participants reported they felt that their placement school staff protected themselves from the sun when outdoors (51.7% agreed, 23.8% strongly agreed) and that there was a clear sun safety culture within the school environment (51.7% agreed, 13.9% strongly agreed).

**TABLE 3:** Experiences and sun behaviours of PSTs while on placement (N = 151)

Question	Strongly disagree n (%)	Disagree n (%)	Neutral n (%)	Agree n (%)	Strongly agree n (%)
*I was briefed on sun safety procedures of the school when I commenced my last school placement*	17 (11.3%)	47 (31.1%)	35 (23.2%)	45 (29.8%)	7 (4.6%)
*On placement, staff protected themselves from sun when outside (eg for outdoor lessons/playground supervision)*	3 (2.0%)	10 (6.6%)	24 (15.9%)	78 (51.7%)	36 (23.8%)
*On placement, I believe there was a clear sun safety message*	1 (0.7%)	16 (10.6%)	46 (30.5%)	67 (44.4%)	21 (13.9%)

Question	Never n (%)	Rarely n (%)	Sometimes n (%)	Often n (%)	Everyday n (%)
*I wore a hat while outside when I was at school on placement*	4 (2.6%)	6 (4.0%)	17 (11.3%)	41 (27.2%)	83 (55.0%)
*I protected myself with sunscreen while outside on placement*	42 (27.8%)	34 (22.5%)	32 (21.2%)	16 (10.6%)	27 (17.9%)

### 3.3 Focus groups and interviews

Throughout the focus groups PSTs provided their opinions on: their attitudes to tanning and barriers and facilitators for using sun protection measures; their experiences with sun safety in Australian primary schools; perceptions of school/teacher support needs; the effect of the PSTSS intervention on personal behaviour and UVR knowledge/awareness; and their intentions to change and future sun safety goals. Themes and sub‐themes generated from the data are shown below.

### 3.4 Personal sun protection attitudes and behaviours

#### 3.4.1 Opinions towards tanning

Many participants in the focus groups and interviews reported that they attempt to tan, particularly in the summer months.

*“I always tan [and] barely ever use sunscreen… I put it on, but [don't remember] to reapply”(FG1)*.

This was not universal however with others reporting they never attempt to tan.

*“I never really go out of my way to go get a tan, I don't really see anything really healthy about it” (INT1_F)*.

Most of the participants who reported tanning were aware of the potential risks associated with this although others did not perceive they personally were at risk.

*“It always tends to be the ‘it‐won't‐happen‐to‐me’ kind of thing. Like, yeah, I get a tan, but I'm not at risk, or anything like that” (FG1)*.

Many participants justified attempting to get a tan by reporting that they feel healthier with a tan.

*“…you're healthier, with a bit of a glow” (FG3)*.

Skin sensitivity to sunburn was reported as a deterrent for attempting to tan with some reporting that their skin would burn rather than tan when exposed to the sun.

#### 3.4.2 Barriers for using sun protection

Use of sun protection was commonly context or temporally based. For example, many PSTs reported that they wore sunscreen while at the beach, at school on placement, or only during the summer months, but not in other settings or seasons. Lack of time and inconvenience were the most commonly reported barriers for sun protection use. Some participants reported that they only would use sunscreen or hats when they were out in the sun for longer than 30 min.

*“Everyone's sort of busy, and sometimes you just think I'm only going to be out in the sun for a couple of minutes, so I'm not going to bother putting sunscreen on, when I just think I'm going to sit outside for a bit” (FG2)*.

The PSTs also reported impracticality and discomfort with sun protection measures as barriers for use. For example, some participants reported a barrier to wearing sun protective clothing was that they did not like wearing heavy clothing in warmer weather.

*“It's a bit impractical…I'm not going to go out wearing a hat, sunglasses, dressed head to toe completely covered every day…[it's just not] realistic” (FG1)*.

Some participants also reported the financial burden and high costs of sunscreen as a barrier.

A number of PSTs reported that they felt embarrassed to use some sun protection measures around their friends as it is not considered fashionable (eg clothing that covers all their skin, broad‐brimmed hats). As a result of peer‐pressure, some reported their sun protective behaviour varied depending on whether they were with their friends or not; with some mentioning they had unsuccessfully, attempted to change peers' opinions towards sun safety.

*“A lot of my friends don't really have UVR knowledge…a lot of people just don't care…a lot of mates don't really care, they're like, ‘Let's go to the beach midday,’ and I try to tell them, but it's hard to get people to listen sometimes, when they do not want to learn”(INT_M3)*.

#### 3.4.3 Facilitators for sun protection

The act of developing a sun protection routine was a commonly reported factor in facilitating the regular use of sun protection measures. Some reported that the pain and discomfort from previous significant sunburns was a deterrent for going out in the sun unprotected and a motivator for using sun protection in the future. Many reported using sunglasses, hats and clothing more often than sunscreen as it was easier to use, easier to remember and had fewer or lower ongoing financial costs. There was high agreement among PSTs that when they were in a school setting they were more likely to use sun protection measures. Acting as a positive role model for their students and complying with the school sun protection policy and rules were the strongest motivators for using sun protection measures in the school setting and helped them to overcome barriers to use that existed in other contexts.

*“On placement, I do wear a hat, because I'm a rule‐abiding citizen. So, these are the things I need to do, to set a good example for the kids, so then I do that. But it's actually almost more of a thing about setting a good example, than actually making sure to implement it for my health, which I'm just realising” (INT_F1)*.

### 3.5 Attitudes towards school approaches

Focus group participants reported that almost all of the schools in which they completed placement had some form of sun protection recommendation in place. However, most reported they never saw the written policies and were unaware of the policy content. Many observed that compliance to hat wearing was the most common method of sun protection which was closely monitored by teachers. Some observed that, based on their experience on placements, compliance was higher in schools where a broad‐brimmed hat was part of the school uniform. The majority of participants observed no classroom‐based teaching about UVR and the UVI. Many participants reported that they did not see sunscreen use in the school and sunscreen use was rarely monitored by the teachers. Perceived reasons for this included: lack of time, limited resources/funding for sunscreen and a lack of sunscreen availability in different parts of the school. PSTs consistently reported that, in their view, it was important that teachers role model sun safe behaviour in front of their students, particularly for use of broad‐brimmed hats. There was consensus among PSTs that in their view there was room to improve sun protection practices in Australian primary schools. PSTs recommended more consistent sun protection messages across primary schools so that all schools implement the same policy, procedures and programs. Participants observed that although schools are attempting to implement school sun protection policies and practices, children are not being taught why they need to protect themselves from UVR. PSTs commonly expressed the view the focus needs to be placed on teaching children the health benefits of being sun safe and the effects that harmful UVR overexposure has on developing skin cancer in later life rather than regulating compliance to school rules (eg mandatory hat wearing, without teaching the children why they are wearing a hat). Participants consistently supported the benefits of additional UVR education at a university, school and community level so that there was consistent messaging for children both inside and outside the school.

#### 3.5.1 Teacher support needs

When asked about strategies for improving existing sun safety approaches in schools, posters, visual aids and tailored lesson plans were positively viewed by PSTs. Participants also recommended professional learning focusing on the UVI for both current and future teachers. Many felt primary teachers face many barriers when teaching children about UVR and sun protection. Some speculated this was due to a lack of time, knowledge of skills or teaching resources. While PSTs observed on placement children not complying with school sun protection recommendations or rules, most were optimistic about the potential to improve sun protective practices in Australian schools.

*“Teachers are already struggling…but I feel like there are ways to incorporate it into lessons, especially with sport and PE. But I feel most teachers aren't educated as well about it” (INT_M3)*.

To improve teacher and school approaches to sun protection, PSTs recommended including more UVR education for both pre‐ and in‐service teachers. They suggested this could be implemented through professional learning opportunities at university and in schools complimented by increased access to free online teaching resources. There was unanimous agreement among participants that the PSTSS intervention was valuable for teachers‐in‐training, with all agreeing that PSTs Australia‐wide should receive tailored sun safety training such as the intervention in this study during their teacher education degree.

### 3.6 Effect of intervention

#### 3.6.1 Personal behaviour

Most PSTs reported that participating in the PSTSS intervention had a positive effect on their sun protective behaviours. Some participants reported developing a new daily sun protection routine as a result of the intervention with many reporting that they used the smartphone application to check the UVI on a daily basis to guide their personal sun protective behaviour.

*“I'm starting to look at that UVR index…and I think that's part of the whole thing, trying to change different habits that we all have, even before we go out, outside of our homes, or playing sports, or going to and from places” (FG2)*.

*“…because of the [intervention] in particular…on placement I realised that I had this routine of going and looking at what the UVR rating was going to be, just because I knew I was going to be spending a third of the time outside and then preparing from there: whether or not I needed a hat, or I needed to go a little bit further. Just because of that routine” (FG1)*.

#### 3.6.2 Perceived knowledge, skills and confidence

The majority of participants reported that being involved in the intervention improved their knowledge and awareness of UVR and the recommended sun protective measures for themselves and their future students. Most participants felt the intervention made them more knowledgeable and skilled to teach sun safety when they enter schools in the future.

*“the information that was given to us [in the intervention], just blew our minds, because there were things that we didn't think of…well, weren't aware of” (FG3)*.

*“I'm more confident in the knowledge of the facts” (FG1)*.

#### 3.6.3 Attitudes towards sun safety education

There was unanimous agreement among participants that the intervention increased their awareness of the importance of sun education for PSTs, in‐service teachers and children.

*“It changed my perceptions of the dangers of harmful UV's” (FG3)*.

*“I think everybody should learn about sun safety in this detail, regardless of their profession…I think it's so important, especially with how high the statistics are… It's baffling that it's not taught” (FG3)*.

#### 3.6.4 Intentions to change and future sun safety goals

Some participants reported that although the intervention did not change their current sun protective behaviours, they intend to improve them in the future, particularly when they move into paid teaching‐related employment.

*“…when I'm a teacher and I've got all those kids every day…I'll need to wear my hat, wear my sunscreen and eventually I feel like it will just become an everyday sort of thing, more of a habit” (FG1)*.

As well as goals specific to the school context, participants also indicated they had personal goals to increase sun protective behaviours to reduce future risks of skin cancer and improve health. These goals related to a range of behaviours including: more frequent hat and sunscreen use; change to using broad‐brimmed hats; long sleeved clothing; daily moisturisers that contain sunscreen; using an app that provide UVR levels and checking the UVI more frequently. Some participants reported that they had good intentions to improve their sun protection practices but ongoing motivation and maintenance was questioned.

*“I definitely want to get better at [protecting myself], because I don't want to die of skin cancer…but it's just whether I can be bothered” (FG3)*.

## 4 DISCUSSION

This exploratory study aimed to investigate PSTs perceptions of the PSTSS intervention and explore their sun behaviours and attitudes towards sun protection and experiences of sun safety approaches in primary schools while on placement. Participants found the brief intervention useful and reflected that additional support teachers to implement UVR and UVI curriculum increased their self‐efficacy to teach sun safety. Being a positive role model and meeting the requirements of school policies were important motivators influencing participants' intentions to use personal sun safety measures when in their teaching role.

Recognising that skin cancer is one of Australia's most preventable cancers, Cancer Council launched the SunSmart Schools program in WA primary and secondary schools in 1998, and later early childhood education and care services in 2005, extending to include participation in all Australian states and territories by 2006 launched.^26^ The program promotes sun protection policies and practices in Australian schools from foundation years to year 12 and aims to minimise harmful UV exposure in school settings.^27^ Recent research indicates that early childcare centres that are members of the SunSmart program enact significantly more sun protection practices compared non‐members.^28^

However, school leaders remain responsible for the extent to which the sun protection programs are implemented in their schools, with varying levels of mandatory policy requirements between jurisdictions from governing bodies such as departments of education and independent educational institutions. In addition, research indicates that the enactment of existing SunSmart primary school policies could be vastly improved.^29^ For skin cancer prevention programs to be effective, schools need to implement sun protection policies, environmental changes and comprehensive educational programs.^30^ Environmental and policy changes can include school guidelines on use of sun protective clothing, hats, sunscreen and shade or restrictions on outdoor activities during peak UVR times. Educational programs should also include comprehensive UVR/UVI curriculum and education for both teachers and students. The level of in‐service teachers understanding of UVR and the UVI remains unknown and should be the focus for future research.

In this study many PSTs reported that in their personal lives, they attempted to get a tan in the summer months even while knowing that this increased their risk of developing skin cancer in later life. Consistent with previous findings, skin sensitivity to sunburn was a deterrent for attempting to tan.^17^, ^31^ PSTs reported many barriers for utilising sun protection measures outside school settings including: discomfort, impracticality, appearance and embarrassment. This may reflect the relatively young age of our sample (three‐quarters were aged ≤24), as comparatively, Australian adolescents have also been reported to positively view sun exposure and negative view sun protection measures with most selecting sunglasses, hats and clothing based on fashion rather than effectiveness of protection.^32^

Interestingly, PSTs reported no issue with wearing wide‐brimmed hats, long sleeves and sunscreen in school settings and all agreed that it was important that teachers model effective sun protection behaviours to their students. Teachers modelling sun safe behaviours has been shown to positively affect students' sun protection behaviours.^33^ Many reported that this was the primary reason for wearing sun protection while in primary schools, rather than being conscious of reducing their own skin cancer risks. Demographic data indicated that the majority of participants in this study were ≤24 years of age and attended school in Australia. Given that the SunSmart School program started in WA in 1998,^27^ it is therefore likely that most would have attended primary schools when these programs were being implemented. We can speculate this may be an influencing factor as to why most PSTs felt motivated to be a role model and comply with school policy in primary school settings. While many reported that there was a clear sun safety message in their placement school, only one third indicated that they were briefed on the sun safety policy on entering the school. Findings indicate a need for schools to comprehensively inform the school community (including visiting staff and PSTs) of existing sun safety policies.

Many study participants reported they saw little‐to‐no UVR or UVI education during their placement. The UVI has been reported to be a valuable tool in determining when protection of the skin from UVR is recommended.^6^ However, Australian research suggests that population understanding and utilisation of the UVI remains minimal.^15^ A recent systematic review of 31 studies from USA, Canada, Europe, Australia, New Zealand and other countries also confirmed that internationally, comprehension of the UVI to inform sun protective behaviours was low.^34^ Many participants viewed the Cancer Council's SunSmart application for mobile devices positively^20^ and some reported they used this as a tool to guide sun protective behaviours. Smartphone application technology which provides simple and easy to read UVI levels alongside sun protective recommendations may be a potential strategy to improve PSTs sun behaviours.^35^ A recent study found that consulting the UVI regularly assisted young people to make decisions about sun protection behaviours.^7^ Further research focusing on the use of the SunSmart application in primary school settings is warranted.

PSTs speculated that practicing teachers faced many barriers when attempting to protect themselves and their students from the sun with many recommending a strength‐based approach (ACARA^36^) to increase children's sun safety skills rather than focusing on sun protection compliance management. PSTs felt educating students to independently use the UVI to inform sun protection practices was a more feasible longer‐term strategy to prevent UVR overexposure in children; but were conscious that teachers need to have the skills and resources to effectively teach their students. Enriching initial teacher education programs in combination with increasing education departments' level of support to enhance schools and teacher's ability to implement holistic health promotion strategies that combine policy, environmental and curriculum approaches to sun safety could be a potential strategy to increase individual's ability to protect themselves from the sun and may minimise many of the reported barriers teachers face.

Participants reported that they found the PSTSS intervention increased their knowledge and awareness of the UVI and risks associated with skin cancer. Consistent with previous findings by Scott et al^37^ participants reported they felt more knowledgeable, skilled and confident to teach sun safety and that the intervention was helpful in increasing their awareness of the UVI as a useful tool to protect themselves from the sun. However, a number also indicated that the brief intervention highlighted gaps in their skills to teach this content in school settings which reduced their confidence levels. While the PSTSS intervention shows promise as a feasible strategy to raise knowledge and awareness of the UVI among PSTs, these findings may indicate that a longer‐term multifaceted approach to school sun safety may be required to develop pre‐ and in‐service teachers skills as such approaches have been shown to be more effective that brief one‐off interventions.^38^ There was unanimous agreement among the PSTs that all tertiary teacher education programs should include comprehensive UVR education so that they can develop the knowledge and skills to properly protect themselves and their future students in school settings. Many PSTs also reported that as a result of attending the PSTSS intervention, they either had changed or intended to change their sun protective behaviours. These findings suggest that some PSTs may in a contemplation, preparation or action phase of behaviour change^39^ which could mean the university setting is an important setting for skin cancer prevention interventions for PSTs.

To the authors' knowledge, this was the first study to explore PSTs perceptions and experiences of sun safety in primary schools when on placement during their initial teacher education degree. A strength of this study was that it used both quantitative and qualitative methodology so as to provide a deeper understanding of participants' personal attitudes behaviours and motivations, as well as their perceptions and experiences of sun safety in primary schools. While this study had noted strengths, there are some limitations. First, this was a exploratory pilot study with a relatively homogeneous sample from one tertiary institution, which may limit the generalisability of findings. Future research studies with samples from a range of different universities with diverse samples is recommended. Second, as this study was exploratory pilot to inform a future larger study, there was no control group. Third, the focus group questions were not pilot tested. Fourth, the focus groups interviews were conducted by members of the research team, who also conducted the intervention, which potentially could have resulted in some participant bias.

## 5 CONCLUSION

There was consensus among PSTs that sun protection in Australian primary schools needs to be improved to maximise the protection of children from harmful UVR overexposure. Participant recommendations included more consistent approaches to sun protection across Australian primary schools with a greater focus on UVR and UVI education, rather than compliance management. Furthermore, innovating and enriching existing teacher education curriculum and programs to include more rigorous content and pedagogical approaches to sun protection, UVR and the UVI education is novel and could support PSTs self‐efficacy to deliver sun safety to students. Based on initial responses to this intervention, such an approach in teacher education programs is likely to be well‐received by PSTs. However, to be effectively implemented such approaches will need to be supported by universities, school policies and be endorsed by governing bodies, school administrators and school communities. Findings from this study provide important information to inform university‐based teacher education interventions for PSTs. Further research testing the feasibility and effectiveness of the interventions such as the PSTSS across different universities and countries is needed.

## AUTHOR CONTRIBUTIONS

All authors are responsible for reported research. J.J.S., R.S.J., J.D., S.B. and M.S. participated in the concept and design, data collection. J.J.S., R.S.J., J.D., S.B., M.S. and B.M. contributed to the analysis, interpretation of data, drafting/revising the manuscript and have approved this manuscript as submitted.

## CONFLICT OF INTEREST

The authors declare no conflict of interest.
